# Attitudes towards mentally ill in Europe (Ukraine)

**DOI:** 10.1192/j.eurpsy.2026.10400

**Published:** 2026-07-31

**Authors:** I. Pinchuk

**Affiliations:** Institute of Psychiatry, Taras Shevchenko National University, Kyiv, Ukraine

## Abstract

**Abstract:**

Attitudes toward people with mental disorders in Ukraine reflect a complex interplay of historical, cultural, and social factors. For decades, psychiatric care remained predominantly institutional: patients were placed in specialised hospitals, which reinforced their isolation and contributed to persistent stigma. This model entrenched the perception of individuals with mental illness as a group incapable of social integration.

Recent healthcare reforms aim to transform this system. The national concept for the development of psychiatric care until 2030 envisions a shift from institutional care to community-based services, as well as the expansion of outpatient centers and mobile teams. Nevertheless, public prejudice remains widespread, manifesting in discrimination in employment, limited access to education, and insufficient social support.

The ongoing war has significantly altered the context of mental health. Widespread traumatic experiences, forced displacement, and the loss of loved ones have led to an increase in cases of post-traumatic stress disorder, anxiety, and depressive conditions. Under these circumstances, societal attitudes toward people with mental illness become not only a matter of human rights but also a factor of national resilience.

Despite persistent challenges, positive trends can be observed: the role of psychologists and social workers is strengthening, attention to patients’ rights is increasing, and mental health is increasingly recognised as an essential component of overall well-being. Ukraine is currently undergoing a transition from the legacy of an institutional model to a modern approach focused on integration and respect for human dignity.

**Disclosure of Interest:**

None Declared

